# 5-Fluoro-2-(4-fluoro­phen­yl)-7-methyl-3-phenyl­sulfinyl-1-benzo­furan

**DOI:** 10.1107/S1600536813017583

**Published:** 2013-06-29

**Authors:** Pil Ja Seo, Hong Dae Choi, Uk Lee

**Affiliations:** a Department of Chemistry, Dongeui University, San 24 Kaya-dong, Busanjin-gu, Busan 614-714, Republic of Korea; b Department of Chemistry, Pukyong National University, 599-1 Daeyeon 3-dong, Nam-gu, Busan 608-737, Republic of Korea

## Abstract

In the title compound, C_21_H_14_F_2_O_2_S, the dihedral angles between the mean plane [r.m.s. deviation = 0.007 (2) Å] of the benzo­furan ring system and the pendant 4-fluoro­phenyl and phenyl rings are 5.93 (9) and 80.23 (5)°, respectively. In the crystal, mol­ecules are linked by weak C—H⋯O and C—H⋯π inter­actions, forming a three-dimensional network.

## Related literature

For background information and the crystal structures of related compounds, see: Choi *et al.* (2011 ▶, 2012 ▶); Seo *et al.* (2011 ▶).
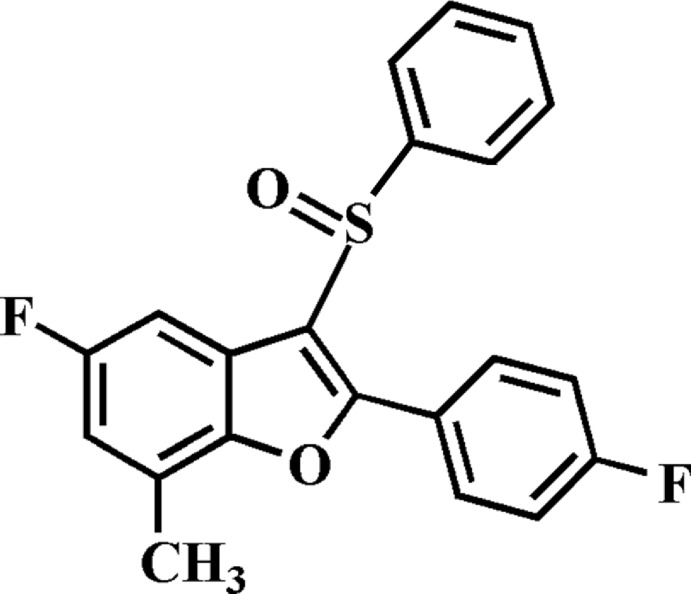


## Experimental

### 

#### Crystal data

C_21_H_14_F_2_O_2_S
*M* *_r_* = 368.38
Monoclinic, 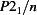

*a* = 12.3698 (8) Å
*b* = 7.9967 (5) Å
*c* = 17.4195 (10) Å
β = 100.323 (4)°
*V* = 1695.20 (18) Å^3^
*Z* = 4
Mo *K*α radiation
μ = 0.22 mm^−1^
*T* = 173 K
0.30 × 0.26 × 0.10 mm

#### Data collection

Bruker SMART APEXII CCD diffractometer
Absorption correction: multi-scan (*SADABS*; Bruker, 2009 ▶) *T*_min_ = 0.655, *T*_max_ = 0.746
30145 measured reflections
4267 independent reflections
3147 reflections with *I* > 2σ(*I*)
*R*_int_ = 0.065

#### Refinement

*R*[*F* ^2^ > 2σ(*F* ^2^)] = 0.044
*wR*(*F* ^2^) = 0.123
*S* = 1.04
4267 reflections
236 parameters
H-atom parameters constrained
Δρ_max_ = 0.34 e Å^−3^
Δρ_min_ = −0.29 e Å^−3^

### 

Data collection: *APEX2* (Bruker, 2009 ▶); cell refinement: *SAINT* (Bruker, 2009 ▶); data reduction: *SAINT*; program(s) used to solve structure: *SHELXS97* (Sheldrick, 2008 ▶); program(s) used to refine structure: *SHELXL97* (Sheldrick, 2008 ▶); molecular graphics: *ORTEP-3* (Farrugia, 2012 ▶) and *DIAMOND* (Brandenburg, 1998 ▶); software used to prepare material for publication: *SHELXL97*.

## Supplementary Material

Crystal structure: contains datablock(s) global, I. DOI: 10.1107/S1600536813017583/gg2118sup1.cif

Structure factors: contains datablock(s) I. DOI: 10.1107/S1600536813017583/gg2118Isup2.hkl

Supplementary material file. DOI: 10.1107/S1600536813017583/gg2118Isup3.cml

Additional supplementary materials:  crystallographic information; 3D view; checkCIF report

## Figures and Tables

**Table 1:** Hydrogen-bond geometry (Å, °) *Cg*1 is the centroid of the C16–C21 phenyl ring.

*D*—H⋯*A*	*D*—H	H⋯*A*	*D*⋯*A*	*D*—H⋯*A*
C20—H20⋯O2^i^	0.95	2.35	3.252 (3)	158
C9—H9*B*⋯*Cg*1^ii^	0.98	2.79	3.519 (2)	132

Symmetry codes: (i) 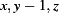; (ii) 
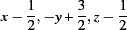
.

**Fig. 1.**
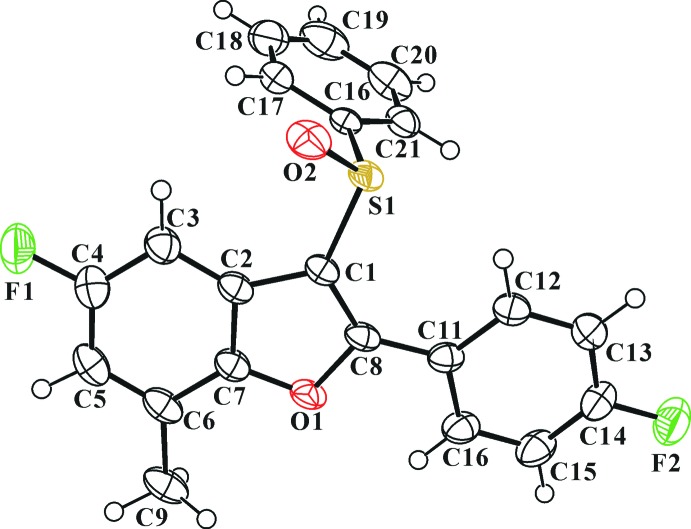
The molecular structure of the title compound with the atom numbering scheme. Displacement ellipsoids are drawn at the 50% probability level. H atoms are presented as small spheres of arbitrary radius.

**Fig. 2.**
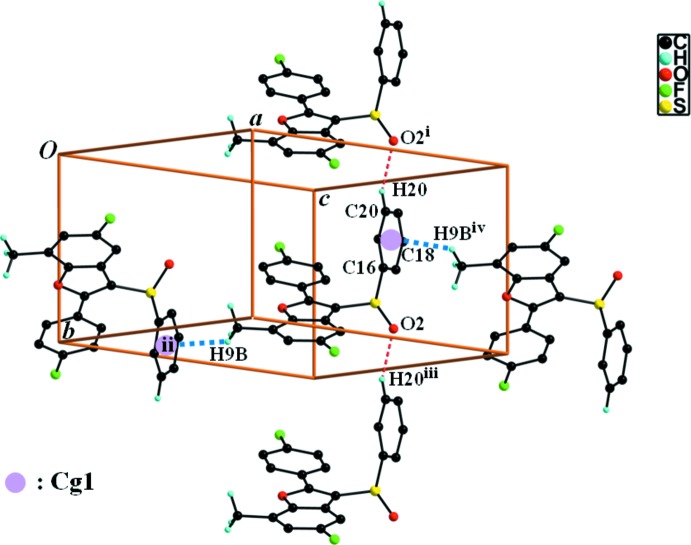
A view of the C–H..O and C–H···π interactions (dotted lines) in the crystal structure of the title compound. H atoms non-participating in hydrogen-bonding were omitted for clarity. [Symmetry codes: (i) x, y - 1, z; (ii) x - 1/2, - y + 3/2, z - 1/2; (iii) x, y + 1, z; (iv) x + 1/2, - y + 3/2, z + 1/2.]

## supplementary crystallographic information

### Comment

As a part of our continuing study of 2-(4-fluorophenyl)-3-phenylsulfinyl-1-benzofuran derivatives containing chloro (Choi *et al.*, 2011), bromo (Seo *et al.*, 2011) and iodo (Choi *et al.*, 2012) substituents in 5-position, we report herein the crystal structure of the title compound.

In the title molecule (Fig. 1), the benzofuran unit is essentially planar, with a mean deviation of 0.007 (2) Å from the least-squares plane defined by the nine constituent atoms. The dihedral angles between the mean plane of the benzofuran ring system and the pendant 4-fluorophenyl and phenyl rings are 5.93 (9) and 80.23 (5)°, respectively. In the crystal structure (Fig. 2), molecules are connected by weak C–H···O and C–H···π interactions (Table 1, Cg1 is the centroid of the C16-C21 phenyl ring), forming a three-dimensional network.

### Experimental

3-Chloroperoxybenzoic acid (77%, 202 mg, 0.9 mmol) was added in small portions to a stirred solution of 5-fluoro-2-(4-fluorophenyl)-7-methyl-3-phenylsulfanyl-1-benzofuran (282 mg, 0.8 mmol) in dichloromethane (30 mL) at 273 K. After being stirred at room temperature for 5h, the mixture was washed with saturated sodium bicarbonate solution and the organic layer was separated, dried over magnesium sulfate, filtered and concentrated at reduced pressure. The residue was purified by column chromatography (benzene) to afford the title compound as a colorless solid [yield 54%, m.p. 466-467 K; *R*_f_ = 0.42 (benzene)]. Single crystals suitable for X-ray diffraction were prepared by slow evaporation of a solution of the title compound in benzene at room temperature.

### Refinement

All H atoms were positioned geometrically and refined using a riding model, with C–H = 0.95 Å for aryl and 0.98Å for methyl H atoms. *U*_iso_(H) = 1.2*U*_eq_(C) for aryl and 1.5*U*_eq_(C) for methyl H atoms. The positions of methyl hydrogens were optimized rotationally.

### Crystal data

**Table:**

C_21_H_14_F_2_O_2_S	*F*(000) = 760
*M**_r_* = 368.38	*D*_x_ = 1.443 Mg m^−^^3^
Monoclinic, *P*2_1_/*n*	Melting point = 466–467 K
Hall symbol: -P 2yn	Mo *K*α radiation, λ = 0.71073 Å
*a* = 12.3698 (8) Å	Cell parameters from 6581 reflections
*b* = 7.9967 (5) Å	θ = 2.2–27.5°
*c* = 17.4195 (10) Å	µ = 0.22 mm^−^^1^
β = 100.323 (4)°	*T* = 173 K
*V* = 1695.20 (18) Å^3^	Block, colourless
*Z* = 4	0.30 × 0.26 × 0.10 mm

### Data collection

**Table:**

Bruker SMART APEXII CCD diffractometer	4267 independent reflections
Radiation source: rotating anode	3147 reflections with *I* > 2σ(*I*)
Graphite multilayer monochromator	*R*_int_ = 0.065
Detector resolution: 10.0 pixels mm^-1^	θ_max_ = 28.5°, θ_min_ = 1.9°
φ and ω scans	*h* = −16→16
Absorption correction: multi-scan (*SADABS*; Bruker, 2009)	*k* = −10→10
*T*_min_ = 0.655, *T*_max_ = 0.746	*l* = −23→23
30145 measured reflections	

### Refinement

**Table:**

Refinement on *F*^2^	Primary atom site location: structure-invariant direct methods
Least-squares matrix: full	Secondary atom site location: difference Fourier map
*R*[*F*^2^ > 2σ(*F*^2^)] = 0.044	Hydrogen site location: difference Fourier map
*wR*(*F*^2^) = 0.123	H-atom parameters constrained
*S* = 1.04	*w* = 1/[σ^2^(*F*_o_^2^) + (0.0545*P*)^2^ + 0.6509*P*] where *P* = (*F*_o_^2^ + 2*F*_c_^2^)/3
4267 reflections	(Δ/σ)_max_ = 0.001
236 parameters	Δρ_max_ = 0.34 e Å^−^^3^
0 restraints	Δρ_min_ = −0.29 e Å^−^^3^

### Special details

**Table:**

Geometry. All esds (except the esd in the dihedral angle between two l.s. planes) are estimated using the full covariance matrix. The cell esds are taken into account individually in the estimation of esds in distances, angles and torsion angles; correlations between esds in cell parameters are only used when they are defined by crystal symmetry. An approximate (isotropic) treatment of cell esds is used for estimating esds involving l.s. planes.
Refinement. Refinement of F^2^ against ALL reflections. The weighted R-factor wR and goodness of fit S are based on F^2^, conventional R-factors R are based on F, with F set to zero for negative F^2^. The threshold expression of F^2^ > 2sigma(F^2^) is used only for calculating R-factors(gt) etc. and is not relevant to the choice of reflections for refinement. R-factors based on F^2^ are statistically about twice as large as those based on F, and R- factors based on ALL data will be even larger.

### Fractional atomic coordinates and isotropic or equivalent isotropic displacement parameters (Å^2^)

**Table:**

	*x*	*y*	*z*	*U*_iso_*/*U*_eq_	
S1	0.75286 (4)	0.76155 (6)	0.66805 (2)	0.02982 (13)	
F1	0.88756 (12)	1.08879 (19)	0.40581 (8)	0.0604 (4)	
F2	0.24627 (11)	0.3963 (2)	0.67822 (8)	0.0594 (4)	
O1	0.52531 (10)	0.79604 (17)	0.47682 (6)	0.0331 (3)	
O2	0.82873 (12)	0.90671 (18)	0.68234 (8)	0.0421 (4)	
C1	0.66892 (15)	0.7947 (2)	0.57601 (9)	0.0293 (4)	
C2	0.70298 (15)	0.8786 (2)	0.51067 (9)	0.0308 (4)	
C3	0.79965 (17)	0.9521 (3)	0.49652 (11)	0.0368 (4)	
H3	0.8643	0.9575	0.5352	0.044*	
C4	0.79512 (19)	1.0161 (3)	0.42283 (12)	0.0420 (5)	
C5	0.70310 (19)	1.0131 (3)	0.36455 (11)	0.0426 (5)	
H5	0.7061	1.0612	0.3151	0.051*	
C6	0.60661 (18)	0.9404 (3)	0.37787 (10)	0.0372 (5)	
C7	0.61135 (16)	0.8754 (2)	0.45189 (10)	0.0317 (4)	
C8	0.56166 (15)	0.7471 (2)	0.55269 (9)	0.0295 (4)	
C9	0.50397 (19)	0.9291 (3)	0.31700 (11)	0.0468 (6)	
H9A	0.4979	0.8165	0.2944	0.070*	
H9B	0.5074	1.0114	0.2759	0.070*	
H9C	0.4397	0.9519	0.3411	0.070*	
C10	0.48089 (15)	0.6561 (2)	0.58759 (10)	0.0310 (4)	
C11	0.49926 (16)	0.6069 (3)	0.66553 (11)	0.0388 (5)	
H11	0.5674	0.6330	0.6980	0.047*	
C12	0.42033 (17)	0.5211 (3)	0.69639 (12)	0.0433 (5)	
H12	0.4332	0.4885	0.7497	0.052*	
C13	0.32297 (17)	0.4839 (3)	0.64853 (12)	0.0412 (5)	
C14	0.30143 (17)	0.5288 (3)	0.57126 (12)	0.0448 (5)	
H14	0.2335	0.5001	0.5392	0.054*	
C15	0.37990 (16)	0.6160 (3)	0.54113 (11)	0.0374 (5)	
H15	0.3654	0.6496	0.4880	0.045*	
C16	0.83023 (15)	0.5900 (2)	0.64087 (9)	0.0285 (4)	
C17	0.92867 (16)	0.6178 (3)	0.61585 (11)	0.0404 (5)	
H17	0.9549	0.7281	0.6105	0.049*	
C18	0.9876 (2)	0.4805 (4)	0.59885 (13)	0.0560 (7)	
H18	1.0550	0.4967	0.5809	0.067*	
C19	0.9509 (2)	0.3218 (4)	0.60738 (13)	0.0593 (7)	
H19	0.9931	0.2291	0.5957	0.071*	
C20	0.8531 (2)	0.2952 (3)	0.63286 (12)	0.0502 (6)	
H20	0.8274	0.1846	0.6382	0.060*	
C21	0.79281 (17)	0.4303 (2)	0.65049 (10)	0.0346 (4)	
H21	0.7260	0.4136	0.6691	0.041*	

### Atomic displacement parameters (Å^2^)

**Table:**

	*U*^11^	*U*^22^	*U*^33^	*U*^12^	*U*^13^	*U*^23^
S1	0.0319 (2)	0.0365 (3)	0.01842 (18)	−0.0017 (2)	−0.00255 (15)	−0.00149 (16)
F1	0.0605 (9)	0.0742 (10)	0.0490 (7)	−0.0177 (8)	0.0169 (7)	0.0140 (7)
F2	0.0472 (8)	0.0810 (11)	0.0534 (8)	−0.0180 (7)	0.0181 (6)	−0.0046 (7)
O1	0.0336 (7)	0.0419 (8)	0.0206 (5)	0.0028 (6)	−0.0039 (5)	0.0011 (5)
O2	0.0475 (9)	0.0385 (8)	0.0342 (7)	−0.0110 (7)	−0.0085 (6)	−0.0043 (6)
C1	0.0323 (9)	0.0332 (10)	0.0203 (7)	0.0009 (8)	−0.0009 (7)	0.0005 (6)
C2	0.0355 (10)	0.0335 (10)	0.0220 (7)	0.0032 (8)	0.0010 (7)	−0.0006 (7)
C3	0.0402 (11)	0.0401 (11)	0.0291 (8)	−0.0035 (9)	0.0038 (8)	0.0001 (8)
C4	0.0492 (13)	0.0424 (12)	0.0364 (10)	−0.0045 (10)	0.0131 (9)	0.0019 (9)
C5	0.0597 (14)	0.0422 (12)	0.0262 (8)	0.0027 (11)	0.0089 (9)	0.0048 (8)
C6	0.0512 (12)	0.0358 (11)	0.0226 (8)	0.0083 (9)	0.0009 (8)	−0.0005 (7)
C7	0.0365 (10)	0.0335 (10)	0.0237 (8)	0.0025 (8)	0.0017 (7)	−0.0010 (7)
C8	0.0319 (9)	0.0346 (10)	0.0195 (7)	0.0048 (8)	−0.0020 (6)	−0.0018 (7)
C9	0.0606 (15)	0.0510 (13)	0.0233 (8)	0.0095 (11)	−0.0073 (9)	0.0016 (8)
C10	0.0282 (9)	0.0351 (10)	0.0282 (8)	0.0040 (8)	0.0010 (7)	−0.0048 (7)
C11	0.0316 (10)	0.0525 (13)	0.0297 (9)	−0.0033 (9)	−0.0020 (7)	0.0011 (8)
C12	0.0386 (12)	0.0578 (14)	0.0330 (9)	−0.0011 (10)	0.0050 (8)	0.0044 (9)
C13	0.0334 (11)	0.0481 (13)	0.0445 (11)	−0.0044 (9)	0.0135 (9)	−0.0064 (9)
C14	0.0311 (11)	0.0622 (15)	0.0398 (10)	−0.0052 (10)	0.0031 (8)	−0.0127 (10)
C15	0.0307 (10)	0.0519 (13)	0.0276 (8)	0.0014 (9)	−0.0003 (7)	−0.0071 (8)
C16	0.0269 (9)	0.0384 (10)	0.0177 (7)	−0.0006 (8)	−0.0030 (6)	0.0017 (7)
C17	0.0341 (11)	0.0562 (13)	0.0297 (9)	−0.0007 (10)	0.0021 (8)	0.0097 (9)
C18	0.0421 (13)	0.091 (2)	0.0365 (11)	0.0184 (13)	0.0116 (9)	0.0080 (12)
C19	0.0697 (17)	0.0669 (18)	0.0392 (11)	0.0320 (15)	0.0044 (11)	−0.0020 (11)
C20	0.0690 (16)	0.0418 (13)	0.0359 (10)	0.0090 (12)	−0.0012 (10)	−0.0012 (9)
C21	0.0385 (11)	0.0374 (11)	0.0253 (8)	−0.0025 (9)	−0.0010 (7)	0.0021 (7)

### Geometric parameters (Å, º)

**Table:**

S1—O2	1.4855 (14)	C10—C11	1.392 (2)
S1—C1	1.7671 (17)	C10—C15	1.399 (3)
S1—C16	1.7846 (19)	C11—C12	1.379 (3)
F1—C4	1.362 (2)	C11—H11	0.9500
F2—C13	1.354 (2)	C12—C13	1.369 (3)
O1—C8	1.3745 (19)	C12—H12	0.9500
O1—C7	1.375 (2)	C13—C14	1.372 (3)
C1—C8	1.370 (3)	C14—C15	1.374 (3)
C1—C2	1.447 (2)	C14—H14	0.9500
C2—C7	1.385 (2)	C15—H15	0.9500
C2—C3	1.393 (3)	C16—C21	1.378 (3)
C3—C4	1.374 (3)	C16—C17	1.383 (3)
C3—H3	0.9500	C17—C18	1.379 (3)
C4—C5	1.383 (3)	C17—H17	0.9500
C5—C6	1.384 (3)	C18—C19	1.365 (4)
C5—H5	0.9500	C18—H18	0.9500
C6—C7	1.382 (2)	C19—C20	1.378 (4)
C6—C9	1.504 (3)	C19—H19	0.9500
C8—C10	1.454 (3)	C20—C21	1.379 (3)
C9—H9A	0.9800	C20—H20	0.9500
C9—H9B	0.9800	C21—H21	0.9500
C9—H9C	0.9800		
			
O2—S1—C1	106.59 (8)	C11—C10—C8	122.99 (16)
O2—S1—C16	107.00 (9)	C15—C10—C8	118.84 (16)
C1—S1—C16	97.50 (8)	C12—C11—C10	121.16 (18)
C8—O1—C7	107.27 (14)	C12—C11—H11	119.4
C8—C1—C2	107.38 (15)	C10—C11—H11	119.4
C8—C1—S1	127.41 (14)	C13—C12—C11	118.55 (19)
C2—C1—S1	125.21 (14)	C13—C12—H12	120.7
C7—C2—C3	119.46 (16)	C11—C12—H12	120.7
C7—C2—C1	104.94 (16)	F2—C13—C12	118.68 (19)
C3—C2—C1	135.60 (17)	F2—C13—C14	118.93 (19)
C4—C3—C2	115.45 (18)	C12—C13—C14	122.4 (2)
C4—C3—H3	122.3	C13—C14—C15	118.77 (19)
C2—C3—H3	122.3	C13—C14—H14	120.6
F1—C4—C3	117.86 (19)	C15—C14—H14	120.6
F1—C4—C5	117.40 (18)	C14—C15—C10	120.96 (18)
C3—C4—C5	124.7 (2)	C14—C15—H15	119.5
C4—C5—C6	120.34 (18)	C10—C15—H15	119.5
C4—C5—H5	119.8	C21—C16—C17	121.41 (19)
C6—C5—H5	119.8	C21—C16—S1	118.17 (14)
C7—C6—C5	114.88 (18)	C17—C16—S1	120.28 (16)
C7—C6—C9	121.7 (2)	C18—C17—C16	118.0 (2)
C5—C6—C9	123.46 (17)	C18—C17—H17	121.0
O1—C7—C6	124.24 (17)	C16—C17—H17	121.0
O1—C7—C2	110.63 (15)	C19—C18—C17	121.2 (2)
C6—C7—C2	125.12 (19)	C19—C18—H18	119.4
C1—C8—O1	109.77 (15)	C17—C18—H18	119.4
C1—C8—C10	135.78 (15)	C18—C19—C20	120.5 (2)
O1—C8—C10	114.43 (15)	C18—C19—H19	119.8
C6—C9—H9A	109.5	C20—C19—H19	119.8
C6—C9—H9B	109.5	C19—C20—C21	119.5 (2)
H9A—C9—H9B	109.5	C19—C20—H20	120.3
C6—C9—H9C	109.5	C21—C20—H20	120.3
H9A—C9—H9C	109.5	C16—C21—C20	119.5 (2)
H9B—C9—H9C	109.5	C16—C21—H21	120.3
C11—C10—C15	118.17 (18)	C20—C21—H21	120.3
			
O2—S1—C1—C8	−147.60 (17)	S1—C1—C8—C10	−1.9 (3)
C16—S1—C1—C8	102.09 (18)	C7—O1—C8—C1	0.2 (2)
O2—S1—C1—C2	32.64 (19)	C7—O1—C8—C10	−178.35 (15)
C16—S1—C1—C2	−77.66 (17)	C1—C8—C10—C11	6.8 (4)
C8—C1—C2—C7	0.2 (2)	O1—C8—C10—C11	−175.11 (18)
S1—C1—C2—C7	−179.97 (14)	C1—C8—C10—C15	−173.3 (2)
C8—C1—C2—C3	−178.9 (2)	O1—C8—C10—C15	4.8 (3)
S1—C1—C2—C3	0.9 (3)	C15—C10—C11—C12	0.0 (3)
C7—C2—C3—C4	−0.3 (3)	C8—C10—C11—C12	179.87 (19)
C1—C2—C3—C4	178.7 (2)	C10—C11—C12—C13	0.4 (3)
C2—C3—C4—F1	−179.76 (18)	C11—C12—C13—F2	178.5 (2)
C2—C3—C4—C5	0.6 (3)	C11—C12—C13—C14	0.1 (4)
F1—C4—C5—C6	179.68 (19)	F2—C13—C14—C15	−179.3 (2)
C3—C4—C5—C6	−0.7 (4)	C12—C13—C14—C15	−0.8 (4)
C4—C5—C6—C7	0.4 (3)	C13—C14—C15—C10	1.2 (3)
C4—C5—C6—C9	−178.7 (2)	C11—C10—C15—C14	−0.7 (3)
C8—O1—C7—C6	179.07 (18)	C8—C10—C15—C14	179.34 (19)
C8—O1—C7—C2	−0.1 (2)	O2—S1—C16—C21	157.93 (13)
C5—C6—C7—O1	−179.11 (18)	C1—S1—C16—C21	−92.11 (14)
C9—C6—C7—O1	0.0 (3)	O2—S1—C16—C17	−17.86 (15)
C5—C6—C7—C2	−0.1 (3)	C1—S1—C16—C17	92.11 (15)
C9—C6—C7—C2	179.01 (19)	C21—C16—C17—C18	1.5 (3)
C3—C2—C7—O1	179.17 (17)	S1—C16—C17—C18	177.18 (15)
C1—C2—C7—O1	−0.1 (2)	C16—C17—C18—C19	−0.9 (3)
C3—C2—C7—C6	0.0 (3)	C17—C18—C19—C20	0.4 (3)
C1—C2—C7—C6	−179.23 (18)	C18—C19—C20—C21	−0.6 (3)
C2—C1—C8—O1	−0.3 (2)	C17—C16—C21—C20	−1.8 (3)
S1—C1—C8—O1	179.93 (13)	S1—C16—C21—C20	−177.49 (14)
C2—C1—C8—C10	177.8 (2)	C19—C20—C21—C16	1.3 (3)

### Hydrogen-bond geometry (Å, º)

Cg1 is the centroid of the C16-C21 phenyl ring.

**Table:**

*D*—H···*A*	*D*—H	H···*A*	*D*···*A*	*D*—H···*A*
C20—H20···O2^i^	0.95	2.35	3.252 (3)	158
C9—H9*B*···*Cg*1^ii^	0.98	2.79	3.519 (2)	132

Symmetry codes: (i) *x*, *y*−1, *z*; (ii) *x*−1/2, −*y*+3/2, *z*−1/2.
